# Phase Ib evaluation of a self-adjuvanted protamine formulated mRNA-based active cancer immunotherapy, BI1361849 (CV9202), combined with local radiation treatment in patients with stage IV non-small cell lung cancer

**DOI:** 10.1186/s40425-019-0520-5

**Published:** 2019-02-08

**Authors:** Alexandros Papachristofilou, Madeleine M. Hipp, Ute Klinkhardt, Martin Früh, Martin Sebastian, Christian Weiss, Miklos Pless, Richard Cathomas, Wolfgang Hilbe, Georg Pall, Thomas Wehler, Jürgen Alt, Helge Bischoff, Michael Geißler, Frank Griesinger, Karl-Josef Kallen, Mariola Fotin-Mleczek, Andreas Schröder, Birgit Scheel, Anke Muth, Tobias Seibel, Claudia Stosnach, Fatma Doener, Henoch S. Hong, Sven D. Koch, Ulrike Gnad-Vogt, Alfred Zippelius

**Affiliations:** 1 Clinic of Radiotherapy and Radiation Oncology, University Hospital Basel, Basel, Switzerland; 2 CureVac AG, Tübingen, Germany; 3 Hospital of St Gallen, St Gallen and University of Bern, Bern, Switzerland; 4 University Hospital Frankfurt, Frankfurt, Germany; 5 Cantonal Hospital of Winterthur, Winterthur, Switzerland; 6 Hospital Graubünden, Chur, Switzerland; 7 Medical Department, Center for Oncology and Hematology, Wilhelminenspital, Wien, Austria; 8 University Hospital Innsbruck, Innsbruck, Austria; 9 Department of Internal Medicine III, University Medical Center Mainz, Mainz, Germany; 10 Thoraxklinik Heidelberg gGmbH, Heidelberg, Germany; 11 Klinik für Allg Innere Medizin, Onkolologie/ Hämatologie, Gastroenterologie, Infektiologie, Esslingen, Germany; 12 Department Hematology and Oncology, Pius Hospital University, Oldenburg, Germany; 13 Department Internal Medicine-Oncology, Medical Campus University of Oldenburg, Oldenburg, Germany; 14 eTheRNA Immunotherapies NV, Niel, Belgium; 15 Medical Oncology, University Hospital Basel, Basel, Switzerland

**Keywords:** Clinical trial, Hypofractionated radiotherapy, Immunomonitoring, mRNA active cancer immunotherapy, Non-small cell lung cancer, BI1361849, CV9202

## Abstract

**Background:**

Preclinical studies demonstrate synergism between cancer immunotherapy and local radiation, enhancing anti-tumor effects and promoting immune responses. BI1361849 (CV9202) is an active cancer immunotherapeutic comprising protamine-formulated, sequence-optimized mRNA encoding six non-small cell lung cancer (NSCLC)-associated antigens (NY-ESO-1, MAGE-C1, MAGE-C2, survivin, 5T4, and MUC-1), intended to induce targeted immune responses.

**Methods:**

We describe a phase Ib clinical trial evaluating treatment with BI1361849 combined with local radiation in 26 stage IV NSCLC patients with partial response (PR)/stable disease (SD) after standard first-line therapy. Patients were stratified into three strata (1: non-squamous NSCLC, no epidermal growth factor receptor (EGFR) mutation, PR/SD after ≥4 cycles of platinum- and pemetrexed-based treatment [*n* = 16]; 2: squamous NSCLC, PR/SD after ≥4 cycles of platinum-based and non-platinum compound treatment [*n* = 8]; 3: non-squamous NSCLC, EGFR mutation, PR/SD after ≥3 and ≤ 6 months EGFR-tyrosine kinase inhibitor (TKI) treatment [*n* = 2]). Patients received intradermal BI1361849, local radiation (4 × 5 Gy), then BI1361849 until disease progression. Strata 1 and 3 also had maintenance pemetrexed or continued EGFR-TKI therapy, respectively. The primary endpoint was evaluation of safety; secondary objectives included assessment of clinical efficacy (every 6 weeks during treatment) and of immune response (on Days 1 [baseline], 19 and 61).

**Results:**

Study treatment was well tolerated; injection site reactions and flu-like symptoms were the most common BI1361849-related adverse events. Three patients had grade 3 BI1361849-related adverse events (fatigue, pyrexia); there was one grade 3 radiation-related event (dysphagia). In comparison to baseline, immunomonitoring revealed increased BI1361849 antigen-specific immune responses in the majority of patients (84%), whereby antigen-specific antibody levels were increased in 80% and functional T cells in 40% of patients, and involvement of multiple antigen specificities was evident in 52% of patients. One patient had a partial response in combination with pemetrexed maintenance, and 46.2% achieved stable disease as best overall response. Best overall response was SD in 57.7% for target lesions.

**Conclusion:**

The results support further investigation of mRNA-based immunotherapy in NSCLC including combinations with immune checkpoint inhibitors.

**Trial registration:**

ClinicalTrials.gov identifier: NCT01915524.

**Electronic supplementary material:**

The online version of this article (10.1186/s40425-019-0520-5) contains supplementary material, which is available to authorized users.

## Background

Active immunotherapy targeting selected tumor associated antigens (TAAs) aims to improve outcomes by producing antigen-specific cellular and/or humoral immune responses [1], with the aim of controlling tumor growth and prolonging survival. However, several phase III trials using this approach in patients with early-, as well as late-stage non-small cell lung cancer (NSCLC) did not reach their primary endpoint [2, 3]. To date, these immunotherapeutic approaches have mostly targeted single antigens, whereby immune escape of tumor cell populations not expressing a single target antigen may have posed a stumbling block. Insufficient immunogenicity of cancer vaccine formulations, not able to break tolerance towards tumor/self-antigens and other immune escape mechanisms, such as activation of inhibitory immune checkpoints [4] may have contributed to these disappointing results. The success of checkpoint blocking antibodies in the treatment of NSCLC offers a new way to overcome immune escape and holds promise to increase the efficacy of antigen-specific therapies in combination.

RNActive® is an mRNA-based vaccination approach, which uses chemically unmodified, sequence-optimized mRNA to encode TAAs for cancer treatment [5–8]. BI1361849 (CV9202) is an RNActive®-based cancer immunotherapy containing sequence-optimized mRNAs encoding different cancer antigens in free and complexed form with the cationic protein protamine; this facilitates antigen expression and activation of the immune system through interaction with toll-like receptor TLR7, TLR8, and intracellular RNA sensors, essentially conferring self-adjuvanting activity, and subsequently inducing an adaptive cellular and humoral immune response [9].

A previous phase I/IIa study has shown that treatment with RNActive®-derived CV9201 cancer immunotherapy in patients with NSCLC was safe and well tolerated, and immune responses against all five encoded antigens were reported [10]. BI1361849 includes the five antigens encoded by CV9201 (cancer/testis antigen 1B [New York esophageal squamous cell carcinoma, NY-ESO-1], melanoma antigen family C1 [MAGE-C1] and C2 [MAGE-C2], baculoviral inhibitor of apoptosis repeat-containing 5 [survivin], and trophoblast glycoprotein [5T4]), together with the mucin-1 (MUC-1) antigen [7]. MUC-1 is frequently overexpressed in NSCLC, and triggering immune responses to this antigen in other clinical trials in this indication has not raised any safety concerns [2, 11]. NY-ESO-1, MAGE-C1 and MAGE-C2 are highly tumor-specific antigens expressed in up to 30% of NSCLC samples, while survivin, 5T4 and MUC-1 are all detected at low levels in healthy tissues but expressed in more than 90% of NSCLC tumors [7]. Based on the frequency of expression of individual antigens in NSCLC, it is estimated that 99.6% of the patient population will express at least one of the antigens encoded by BI1361849 [12, 13].

Radiotherapy can induce immunogenic cell death and stimulate tumor-specific immune responses, resulting in regression of non-irradiated lesions [14]. However, such abscopal responses observed outside the field of radiation remain relatively rare in the clinic after radiotherapy alone. Nevertheless, the ability of radiotherapy to induce immunogenic cancer cell death and to promote recruitment and function of T cells within the immunosuppressive tumor microenvironment, provides a rationale for combining radiotherapy with immunotherapy [15, 16]. Preclinical data provide evidence for synergy between radiotherapy and active immunotherapy [17–21], and there is also evidence of synergy in the clinical setting, including in metastatic NSCLC [22–26]. While active immunotherapy may support an abscopal effect of radiation, preclinical data suggest that the tumor response varies with the size of radiotherapy dose per fraction [27].

This article describes results from a phase Ib clinical trial that aimed to assess the safety and tolerability of BI1361849 mRNA-based active cancer immunotherapy combined with fractionated local radiation and different standard of care maintenance treatments in patients with stage IV NSCLC.

## Methods

Details of the study design, which have been published previously [7], are summarized below.

### Study design

This was an exploratory, open-label, multicenter, phase Ib study of mRNA-based active cancer immunotherapy BI1361849 (CV9202) and local radiation as consolidation and maintenance treatment in patients with stage IV NSCLC and a response or stable disease (SD) after first-line chemotherapy or therapy with the epidermal growth factor receptor (EGFR) tyrosine kinase inhibitors (TKI) erlotinib or gefitinib (ClinicalTrials.gov identifier: NCT01915524).

The study was performed according to Good Clinical Practice and in line with the Declaration of Helsinki and local regulations.

### Patients

Eligible patients were ≥ 18 years old with histologically or cytologically confirmed stage IV NSCLC and, for those with non-squamous cell histology, a confirmed EGFR mutation status. All patients should have achieved a partial response (PR) or SD according to Response Evaluation Criteria for Solid Tumors (RECIST) version 1.1 after receiving first-line therapy. Patients had to have at least one tumor lesion eligible for radiation, and at least one additional measurable tumor lesion according to RECIST version 1.1 (see further details in Additional file 1: Supplementary methods). Patients also had Eastern Cooperative Oncology Group (ECOG) performance status 0 to 1, and adequate organ function.

Exclusion criteria (considered in more detail in Sebastian et al. 2014 [7]) included previous active immunotherapy for NSCLC (including immunotherapy with anti-CTLA4 antibodies) and an estimated life expectancy of ≤3 months.

**Fig. 1:**
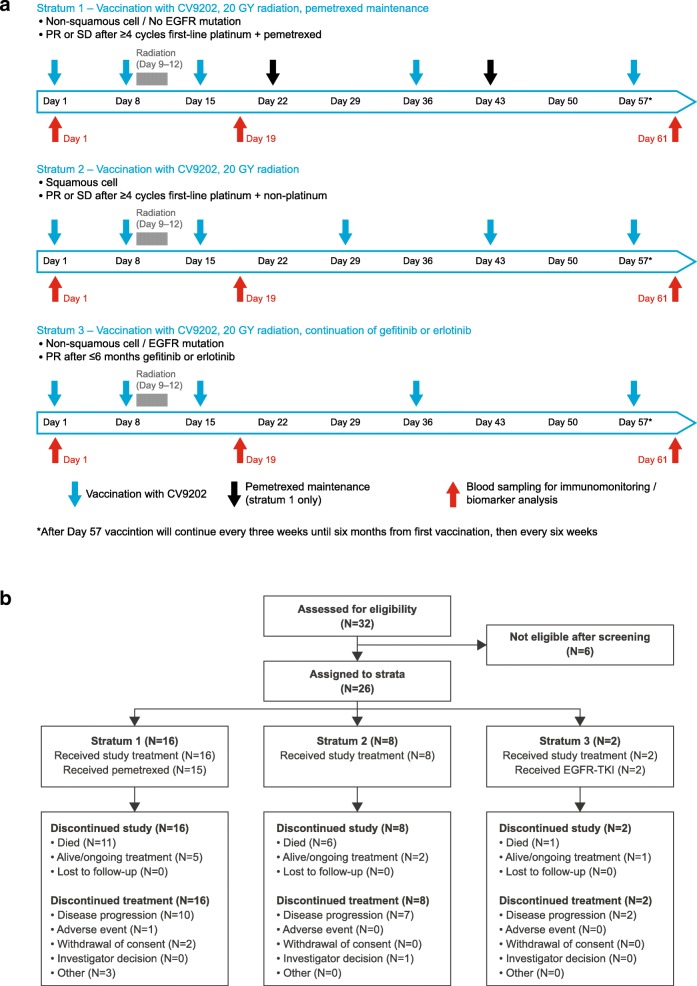
**a** Study design. **b** Patient disposition

Patients were enrolled into one of three study arms (stratum 1, 2, or 3) based on their molecular and histological subtype of NSCLC (Fig. 1a and b). Stratum 1 involved patients with non-squamous NSCLC without activating EGFR mutations, who had PR or SD after at least four cycles of platinum- and pemetrexed-based treatment, and had an indication for maintenance therapy with pemetrexed. Stratum 2 contained patients with squamous NSCLC, who had PR or SD after at least four cycles of platinum-based and non-platinum compound treatment. Stratum 3 comprised patients with non-squamous NSCLC and an activating EGFR mutation, who had PR or SD after at least 3, and up to 6 months of EGFR-TKI treatment.

### Treatment

Patients started screening 2 weeks after their last cycle of first-line chemotherapy (in strata 1 and 2) or within 6 months of starting EGFR-TKI therapy (stratum 3). BI1361849 was administered on study days 1 and 8, followed by radiation therapy on days 9–12 (Fig. 1a). Patients in strata 1 and 3 subsequently received three further treatments with BI1361849, on days 15, 36, and 57, whereas those in stratum 2 received four, on days 15, 29, 43, and 57, after which patients in all three strata were treated with BI1361849 at 3-week intervals for the first 6 months, then every 6 weeks thereafter. Treatment with BI1361849 was continued until disease progression requiring the start of systemic second-line treatment or patients experiencing unacceptable toxicity.

Preclinical data support a vaccination schedule comprising frequent and continued vaccination with an interval of 1 week in the priming phase in combination with a fragmented radiation regimen. The synergistic anti-tumor effect of immunotherapies with radiotherapy has been shown in different preclinical models [17, 18, 20, 21, 28]. Importantly, complete primary tumor regression as well as abscopal effects were only observed in a mouse breast cancer model when radiation was given as a fractionated regimen together with anti-CTLA4 but not when the antibody was given either alone or in combination with single-dose radiotherapy [18].

The vaccination regimen employed in this study is in line with a vaccination regimen used in a trial with CV9201, a precursor mRNA-based lung cancer vaccine [10]. Since expected progression free survival (PFS) was shorter in patients with squamous cell carcinoma (stratum 2) and no concomitant maintenance treatment was administered, a slightly more intense vaccination schedule was applied in this stratum. This more intense schedule is also supported by preclinical data, suggesting that more frequent administrations may enhance the generation of antigen-specific immune responses (unpublished observations). Pre-clinical studies, however, only provide an indication of vaccination efficacy and clinical studies are required to determine the most efficacious treatment regimen for patients.

The recommended dose of BI1361849 was based on the recommended dose per individual mRNA as determined in a phase I/IIa trial of CV9201, a precursor mRNA vaccine (NCT00923312, [10]). At each BI1361849 administration time point, patients received 320 μg mRNA per antigen resulting in a total dose of 1920 μg mRNA. The six components (one per encoded antigen) were administered separately, with patients receiving 2 × 200 μL intradermal injections per component. The 12 injections were distributed across four lymph node targeting areas, i.e. three injections in each of the right and left upper inner arm, as well as in the right and left inner thigh. In addition, patients in stratum 1 received pemetrexed maintenance therapy every 21 days, while erlotinib/gefitinib continuation was available for those in stratum 3.

Radiation therapy was delivered to a dose of 20 Gy over four consecutive days with a daily fraction of 5 Gy. The administration of 4 × 5 Gy represents a well-established palliative radiation regimen that can be applied to pulmonary lesions, bone and soft tissue lesions with low toxicity [29]. Radiation target definitions are described in Additional file 1.

### Study endpoints

The primary endpoint was the number of patients who experienced BI1361849- and/or radiation-related adverse events (AEs) of ≥ grade 3 according to National Cancer Institute–Common Terminology Criteria for Adverse Events (NCI-CTCAE), version 4.0. The pre-defined margin for acceptable safety and tolerability for this endpoint was ≤30% of patients. Secondary safety endpoint evaluation included consideration of other treatment-emergent AEs (TEAEs), as well as clinical and laboratory assessments.

Other secondary endpoints involved assessment of immune responses (as defined in the section below) — consideration of cellular and humoral immune responses to the antigens encoded by the six BI1361849 mRNA components, as well as humoral immune responses to a panel of antigens not covered by the immunotherapeutic (for assessment of ‘broadening of the humoral immune response’).

Secondary efficacy endpoints were tumor response according to RECIST version 1.1 [30], time to start of, and response to, second-line cancer treatment, PFS, and overall survival (OS) from time of first BI1361849 administration.

### Assessments and immunological assays

Safety assessments included monitoring of AEs and hematological and biochemical tests. They are described in detail together with immune response analysis and efficacy assessment in Additional file 1. Based on the prediction that 99.6% of the patient population will express at least one of the antigens encoded by BI1361849, individual patient tumors were not assessed for antigen expression.

#### Intracellular cytokine staining (ICS) and flow cytometry

Antigen-specific T cells were assessed by ICS measuring interferon-γ (IFN-γ), tumor necrosis factor-α (TNF-α) and interleukin-2 (IL-2) production, and CD107a translocation by CD4^+^ and CD8^+^ T cells. Peripheral blood mononuclear cells (PBMCs) were thawed and stimulated ex vivo with overlapping 15-mer peptide libraries each covering the full open reading frame of the six BI1361849-encoded antigens (JPT Peptide Technologies GmbH, Germany) including literature-selected short class-I peptides (Additional file 2: Table S1). Short-term cell culture was performed for 6 h in the presence of anti-CD28 (CD28.2), anti-CD49d (L25), phycoerythrin (PE) anti-CD107a (H4A3) antibodies (BD Biosciences, Germany) and GolgiPlug/GolgiStop (BD Biosciences). Cells stimulated with culture medium X-Vivo 15 (Lonza, Switzerland) and dimethyl sulfoxide (Sigma-Aldrich Chemie GmbH, Germany) served as background controls. In addition, each experimental run included a positive control consisting of PBMCs stimulated with a peptide mix including epitopes from cytomegalovirus, Epstein-Barr virus and flu virus (CEF; JPT Peptide Technologies GmbH, Germany) and Staphylococcus enterotoxin B (SEB) (Sigma-Aldrich Chemie GmbH, Germany). Stimulated PBMCs were incubated with Fc-receptor block (Miltenyi Biotec GmbH, Germany) and labeled with LIVE/DEAD® Fixable Aqua Dead Cell Stain Kit (Thermo Fisher Scientific, USA) and Brilliant Violet650 anti-CD8 (RPA-T8), APC-H7 anti-CD3 (SK7), PE-Cy7 anti-CD4 (MP4-25D2), AlexaFluor700 anti-CD14 (M5E2), or AlexaFluor700 anti-CD19 (HIB19) (BD Biosciences). Cells were fixed and permeabilized using Cytofix/Cytoperm (BD Biosciences) and ICS was performed for the activation marker CD69 (Brilliant Violet605 anti-CD69, clone FN50, BioLegend, Germany) and cytokines using fluorescein isothiocyanate anti-IFN-γ (B27) (BD Biosciences), PerCP-Cy5.5 anti-TNF-α (Mab11), or Brilliant Violet 421 anti-IL-2 (MQ1-17H12) (BioLegend) antibodies.

Immune response analysis included determination of cellular immune responses ex vivo by enzyme-linked immunosorbent spot (ELISpot) assay and intracellular cytokine staining (ICS). Antigen-specific antibodies were assessed via enzyme-linked immunosorbent assay (ELISA). Seromic profiling, using the Serametrix NSCLC-specific antigen microarray, was performed to investigate broadening of humoral immunity. Efficacy was assessed by radiological tumor assessments.

Criteria to identify cellular and humoral immune responses included an at least two-fold increase of either functional T cells or antibody levels over background in addition to empirically determined minimum thresholds (e.g. percentages of functional T cells > 0.1% or at least 10 events in the functional gate). Depending on the specific assay, in the absence of official guidance or recommendations, further empirical rules for definitions were applied, in addition to the aforementioned increases, as described in Additional file 1. In addition, the frequencies of patients were determined with cellular or humoral immune response magnitudes at least two-fold higher compared to baseline (day 1).

### Statistical analysis

A formal sample size calculation was not applicable — a planned sample size of 36 patients was chosen, based on previous observations with predecessor vaccines of BI1361849, whereby a minimum of eight patients per stratum was considered necessary to evaluate the frequency of immune responses.

The safety and efficacy evaluations were performed on all patients who received at least one dose of study medication (safety analysis set [SAF]).

Assessments of immune responses were performed on patients in the SAF who received at least three administrations of BI1361849 and 4 × 5 Gy radiotherapy according to the protocol, had at least one post-vaccine immune sample collected, and did not receive relevant immunosuppressive concomitant medication (i.e. evaluable population). The evaluable population included 25 patients overall.

SAS, GraphPad Prism 6 and Microsoft Excel 2010 were used for statistical data analysis.

Survival data was estimated from the time of treatment initiation using Kaplan-Meier methodology.

## Results

### Patient disposition and characteristics

The study was conducted at 13 centers in Germany, Austria and Switzerland, and enrolled 32 patients; six patients failed screening (see Additional file 3: Table S2) and 26 were assigned to the different strata (Fig. 1b). The first patient was recruited in April 2013; the last patient completed the study in July 2016.

The recruitment goal of eight patients to stratum 3 could not be achieved within a reasonable time frame and recruitment was stopped prematurely, with only two patients enrolled in this stratum. This was because only few patients had eligible lesions for radiation after initial therapy with EGFR-TKIs (erlotinib and gefitinib).

Patients’ baseline characteristics, reflecting those of a stage IV NSCLC population, are shown in Table 1.

**Table 1:** Baseline characteristics of the patient population

Characteristic	Stratum 1 (*n* = 16)	Stratum 2 (*n* = 8)	Stratum 3 (*n* = 2)	Overall (*n* = 26)
Gender, n (%)				
Male	5 (31.3)	7 (87.5)	1 (50.0)	13 (50.0)
Female	11 (68.8)	1 (12.5)	1 (50.0)	13 (50.0)
Age, years				
Median (range)	61.0 (40–74)	70.0 (58–83)	66.0 (59–73)	63.0 (40–83)
ECOG, n (%)				
0	5 (31.3)	0	2 (100.0)	7 (26.9)
1	11 (68.8)	8 (100.0)	0	19 (73.1)
NSCLC duration at baseline, days				
Median (range)	131.0 (99–482)	117.0 (92–762)	167.0 (141–193)	123.5 (92–762)
Previous first-line chemotherapy				
Yes, n (%)	16 (100.0)	8 (100.0)	0	24 (92.3)
No, n (%)	0	0	2 (100.0)	2 (7.7)
Treatment response,^a^ n (%)				
CR	0	0	–	0
PR	9 (56.3)	3 (37.5)	–	12 (46.2)
SD	7 (43.8)	5 (62.5)	–	12 (46.2)
PD	0	0	–	0
NE	0	0	–	0
Previous EGFR-TKI therapy				
Yes, n (%)	0	0	2 (100.0)	2 (7.7)
No, n (%)	16 (100.0)	8 (100.0)	0	24 (92.3)
Treatment response, n (%)	–	–	2 (100.0)^b^	2 (7.7)

*Abbreviations*: *CR* complete response, *ECOG* Eastern Cooperative Oncology Group, *EGFR-TKI* epidermal growth factor receptor tyrosine kinase inhibitor, *NE* not evaluable, *NSCLC* non-small cell lung cancer, *PR* partial response, *SD* stable disease

^a^With respect to previous first-line chemotherapy for NSCLC (strata 1 and 2)

^b^Both patients had partial response

### Treatment exposure and overall safety

The mean number of successful BI1361849 administrations, defined as successful administration of at least 10 of the 12 injections per treatment, was 8.4 (range 2–25). The median duration of BI1361849 treatment was 81 days (range 8–806 days). All patients received a radiation dose of 20 Gy in four fractions of 5 Gy per protocol. Further details of treatment exposure are given in Additional file 4: Table S3.

For the primary study endpoint, BI1361849- and/or radiation- related AEs of ≥grade 3 were reported in four (15.4%) of the 26 patients, overall: two patients (12.5%) in stratum 1 (one event each of dysphagia and fatigue), one patient (12.5%) in stratum 2 (fatigue), and one patient (50.0%) in stratum 3 (pyrexia) had grade 3 events. For strata 1 and 2, these frequencies were below the pre-defined margin of ≤30% of patients; the small sample size of stratum 3 (*n* = 2) did not allow determination of meaningful percentages in this group. Three of the four events (fatigue [two events] and pyrexia) were related to administration of BI1361849, while one (dysphagia) was related to study radiation. There were no serious TEAEs related to BI1361849 and no TEAEs leading to death. No immune-related TEAES were reported.

Table 2 provides an overview of the TEAEs. The most frequently reported TEAEs were injection-site reactions, transient flu-like symptoms or, for stratum 1 only, AEs typically associated with pemetrexed therapy; no immune-related TEAEs were reported. Common TEAEs (occurring in ≥10% of patients overall) are summarized in Additional file 5: Table S4.

**Table 2:** Overview of treatment-emergent adverse events (safety analysis set)

Patients with at least one event, n (%)	Stratum 1 (*n* = 16)	Stratum 2 (*n* = 8)	Stratum 3 (*n* = 2)	Overall (*n* = 26)
TEAE	16 (100.0)	8 (100.0)	2 (100.0)	26 (100.0)
BI1361849- and/or radiation-related AE	16 (100.0)	8 (100.0)	2 (100.0)	26 (100.0)
TEAE related to BI1361849	15 (93.8)	8 (100.0)	2 (100.0)	25 (96.2)
TEAE related to radiation	4 (25.0)	1 (12.5)	0 (50.0)	5 (19.2)
Serious TEAE	7 (43.8)	3 (37.5)	1 (50.0)	11 (42.3)
Serious BI1361849- and/or radiation-related AE	1 (6.3)	0	0	1 (3.8)
Related to BI1361849	0	0	0	0
Related to radiation	1 (6.3)	0	0	1 (3.8)
TEAE toxicity grade ≥ 3^a^	9 (56.3)	4 (50.0)	2 (100.0)	15 (57.7)
BI1361849- and/or radiation-related AE toxicity grade ≥ 3^a^	2 (12.5)	1 (12.5)	1 (50.0)	4 (15.4)
Related to BI1361849	1 (6.3)	1 (12.5)	1 (50.0)	3 (11.5)
Related related to radiation	1 (6.3)	0	0	1 (3.8)
Serious BI1361849- and/or radiation-related AE toxicity grade ≥ 3^a^	1 (6.3)	0	0	1 (3.8)
Related to BI1361849	0	0	0	0
Related to radiation	1 (6.3)	0	0	1 (3.8)
TEAE leading to discontinuation	4 (25.0)	0	0	4 (15.4)
TEAE toxicity grade ≥ 3 leading to discontinuation	2 (12.5)	0	0	2 (7.7)
TEAE leading to interruption/dose modification	4 (25.0)	0	0	4 (15.4)
TEAE leading to death	0	0	0	0

*Abbreviations*: *AE* adverse event, *TEAE* treatment-emergent adverse event

^a^National Cancer Institute–Common Terminology Criteria for Adverse Events (NCI-CTCAE) toxicity grading

Clinically relevant changes in autoimmunity parameters were not reported.

### Humoral and cellular immune assessments

According to the mechanism of action of BI1361849 as an active cancer immunotherapy, pre-existing and post-vaccine immune responses were measured ex vivo without prior expansion by in vitro stimulation. Representative IFN-γ ELISpot results for a patient reacting to the antigen 5T4 are shown in Additional file 6: Figure S1a). Antigen-specific CD4^+^ and CD8^+^ T cells were analyzed by ICS (Representative CD8^+^ analysis in Additional file 6: Figure S1b, c).

**Fig. 2:**
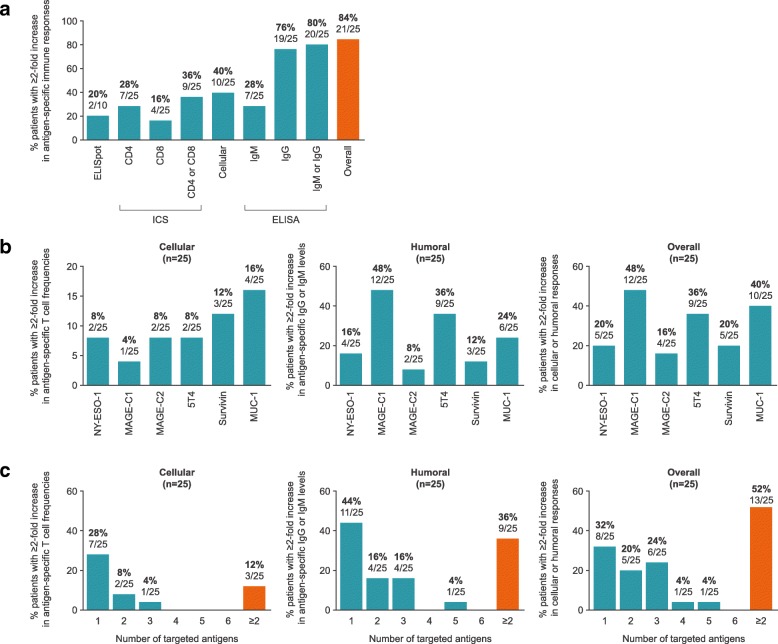
Frequencies of patients with an at least two-fold increase in antigen-specific immune responses following BI1361849 immunotherapy combined with local radiation treatment. Values displayed above the bars indicate the percentages and actual number of patients with increase in immune responses. **a** Summary graph showing frequencies of patients with antigen-specific T cells, antibodies or both exhibiting an at least two-fold increase compared to baseline against one or more antigens encoded by BI1361849 (any post-vaccine time point). CD4 = antigen-specific CD4^+^ T cells, CD8 = antigen-specific CD8^+^ T cells. **b** Frequencies of patients with an at least a two-fold increase in immune responses compared to baseline to each of the antigens encoded by BI1361849 shown as percentage of all evaluable patients; any post-vaccine time point. **c** Frequencies of patients with antigen-specific T cells, antibodies or both showing at least a two-fold increase compared to baseline against multiple antigens encoded by BI1361849 shown as percentage of all evaluable patients; at any post-vaccine time point

Twenty-five patients were evaluable for immunomonitoring, of whom 84.0% (21/25) fulfilled the criteria of exhibiting an at least two-fold increase in immune response magnitude compared to baseline against one or more of the BI1361849 antigens (Fig. 2a). In detail, a total of 10/25 (40%) evaluable patients fulfilled the pre-specified criteria of at least two-fold increased magnitudes of functional CD4^+^ and/or CD8^+^ T cells determined by ICS or ELISpot and 20/25 (80%) met the criteria of a two-fold increased antigen-specific IgM and/or IgG level compared to baseline on at least one post-vaccine time point (Fig. 2a). Patients with at least two-fold increased immune response magnitudes have been detected in all three strata (see Additional file 7: Table S5). Immune responses directed against each of the six antigens encoded by BI1361849 were detected (Fig. 2b), while 52% of patients (13/25) reacted against multiple antigens (Fig. 2c).

**Fig. 3:**
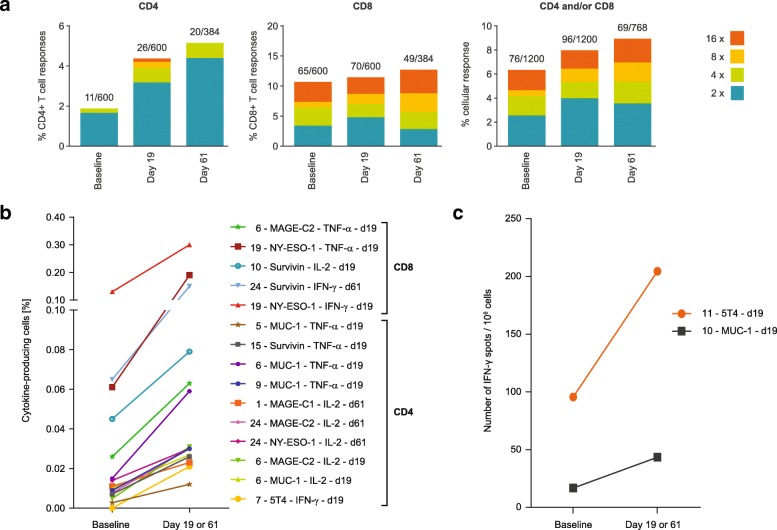
**a** Vaccine antigen-specific CD4^+^ and CD8^+^ T cells detected by ICS at baseline (*n* = 7 patients for panel CD4, *n* = 11 patients for panel CD8, *n* = 16 patients for panel CD4 and/or CD8), and days 19 (*n* = 13 patients for panel CD4, *n* = 15 patients for panel CD8, *n* = 23 patients for panel CD4 and/or CD8) and 61 (*n* = 7 patients for panel CD4, *n* = 7 patients for panel CD8, *n* = 10 patients for panel CD4 and/or CD8) following BI1361849 immunotherapy combined with local radiation treatment. Vaccine antigen-specific T cell responses were determined and are shown as percentages of all possible responses. The denominator for calculating the percentage was defined as the maximum of all possible at least two-fold increases over background (unstimulated cells). Example: 4 functions (IFN-γ, TNF-α, IL-2, CD107a) * 2 T cell subsets (CD4^+^ or CD8^+^ T cells) * 6 BI1361849 antigens * 25 patients = 1200 possible responses. The numbers in the key show fold-increases over the background control. **b** & **c** Magnitude of T cell responses as measured by **b** ICS or **c** IFN-γ ELISpot of patients with an at least two-fold increase in antigen-specific immune responses following BI1361849 treatment. Values are shown as the percentage of positive cells gated on both CD4^+^ or CD8^+^ populations for ICS. ELISpot results are plotted as number of spots per 1 million PBMCs after background subtraction (PBMCs that received only cell culture medium). Legends indicate patient ID, antigen, measured cytokine (only for ICS) and time point

We defined functionality of T cells as their ability to induce antigen-specific immune responses if the magnitude of their response exceeded the background control (PBMCs that have received only cell-culture medium) by at least two-fold. Additional exploratory *post-hoc* analysis of such immune responses showed that the frequencies of functional CD4^+^ and CD8^+^ T cells following BI1361849 immunotherapy combined with radiotherapy increased over time (Fig. 3a). Detectable increases of CD4^+^ and CD8^+^ T cells to BI1361849 antigens post-vaccine were mostly driven by CD4^+^ T cells (Fig. 3a, left-hand panel). The magnitude of the responses to individual antigens detected post-vaccination varied among patients (Fig. 3b and c) with most responses detected by ICS but not ELISpot. In one patient corresponding CD4 and CD8 T cell responses against the same antigen were observed by ICS (patient 6, MAGE-C2). The majority of the T cell responses detected by ICS were mono-functional.

We hypothesized that the combination of radiotherapy with active tumor immunotherapeutic BI136184 can initiate an antigen cascade to broaden the anti-tumor immune response [31]. To address this question, we profiled the antibody repertoire against NSCLC antigens using an antibody array containing 32 known NSCLC TAAs. Broadening of the antibody repertoire against antigens not covered by BI1361849 was observed in 50.0% of all evaluable patients (12/24; any post-vaccine time point; Additional file 8: Figure S2) and in eight of the 14 analyzable pemetrexed treated patients (57.1%) in stratum 1. Some patients had already high numbers of pre-existing antibodies against TAAs. In most cases, such repertoires did not increase during treatment, but were generally maintained.

### Efficacy

**Fig. 4:**
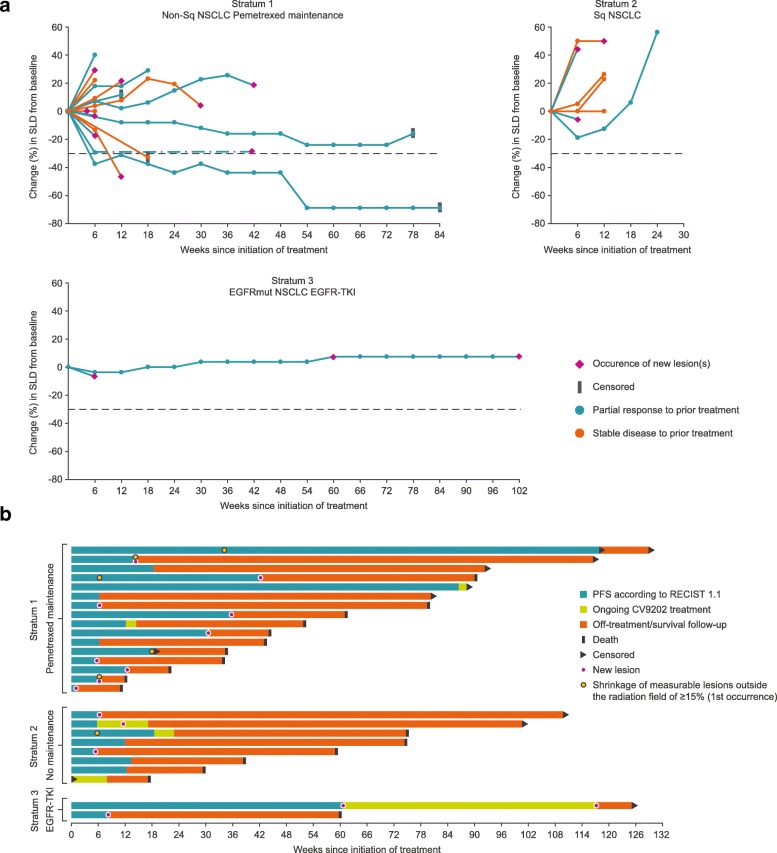
Efficacy outcomes following BI1361849 immunotherapy combined with local radiation treatment. **a** Change in target lesion sum of longest diameters (SLD) from baseline. **b** Survival, progression-free survival, and response kinetics of individual patients (post hoc swimmer plot)

Of the 26 patients in the safety set (SAF) evaluated for efficacy, overall, 46.2% (12/26) demonstrated SD as the best confirmed overall response (Table 3). One patient achieved a confirmed PR with decreasing measurable tumor size up to the last follow-up visit. This patient received concomitant pemetrexed (stratum 1) and had already shown a PR to the previous chemotherapy. Another patient (also from stratum 1) exhibited decreasing target lesion sizes not formally qualifying as a PR. Shrinkage of non-irradiated lesions > 15% occurred in six patients, five in stratum 1, and one in stratum 2. Figure 4a shows the percentage change in target lesion sizes (sum of longest diameters) by stratum. Best overall response according to lesion type (target/non-target) is shown in Additional file 9: Table S6.

**Table 3:** Best overall response (safety analysis set)

Parameter	Patients with response, n (%) [95% confidence interval]			
	Stratum 1 (*n* = 16)	Stratum 2 (*n* = 8)	Stratum 3 (*n* = 2)	Overall (*n* = 26)
Response (CR + PR) rate	1 (6.3) [0.2–30.2]	0 [0.0–36.9]	0 [0.0–84.2]	1 (3.8) [0.1–19.6]
Best overall response				
CR	0 [0.0–20.6]	0 [0.0–36.9]	0 [0.0–84.2]	0 [0.0–13.2]
PR	1 (6.3) [0.2–30.2]	0 [0.0–36.9]	0 [0.0–84.2]	1 (3.8) [0.1–19.6]
SD	8 (50.0) [24.7–75.3]	3 (37.5) [8.5–75.5]	1 (50.0) [1.3–98.7]	12 (46.2) [26.6–66.6]
PD	7 (43.8) [19.8–70.1]	4 (50.0) [15.7–84.3]	1 (50.0) [1.3–98.7]	12 (46.2) [26.6–66.6]
NE	0 [0.0–20.6]	1 (12.5) [0.3–52.7]	0 [0.0–84.2]	1 (3.8) [0.1–19.6]

Confirmed response according to Response Evaluation Criteria for Solid Tumors (RECIST) version 1.1

*Abbreviations*: *CR* complete response, *NE* not evaluable, *PD* progressive disease, *PR* partial response, *SD* stable disease

Second-line cancer treatment was received by 73.1% of patients overall (*n* = 19). The median times to the initiation of second line therapy were 14.4 weeks (range 7–46) in stratum 1, 15.6 weeks (range 7–30) in stratum 2, and 62.6 weeks (range 8–117) in stratum 3. Most patients responded to the second-line cancer treatment with SD or PD (31.6% [6/19] and 36.8% (7/19], respectively]. One patient, treated with crizotinib and trastuzumab emtansine, achieved a PR. Five patients were not evaluable.

Information for the individual patients by stratum is provided in Fig. 4b, which shows a swimmer plot, illustrating PFS time and OS time, in addition to response kinetics. Overall, median PFS was 2.87 months (95% confidence interval [CI] 1.43–4.27) (Additional file 10: Figure S3a). Overall, the median OS time from first BI1361849 treatment was 13.95 months (95% CI 8.93–20.87; Additional file 10: Figure S3b).

## Discussion

To our knowledge, this study is among the first to combine mRNA-based immunotherapy with fractionated local radiotherapy for the treatment of cancer [32]. In our study, BI1361849 (CV9202) a cancer immunotherapy based on sequence-optimized mRNA formulated with protamine was combined with fractionated local radiation treatment in patients with stage IV NSCLC and appeared safe and well tolerated with or without maintenance pemetrexed; only four patients experienced a grade 3 BI1361849- and/or radiation-related AE and there was no evidence of autoimmune events. Based on only two patients enrolled in the stratum with EGFR-TKIs, no definitive conclusion on safety of this combination is possible, but there was no overt evidence of increased toxicity.

As we did not observe occurrence of radiation pneumonitis in our study, testing of BI1361849 in combination with fractionated radiotherapy and pemetrexed therapy in further trials is supported.

The immunomonitoring results appear comparable to those observed previously with the protamine formulated precursor vaccine CV9201 alone without radiation in a phase IIa study in patients with NSCLC where antigen-specific immune reactions against at least one antigen were detected in 63% of evaluable patients [10]. However, differences in study methodologies, e.g. sample time points, and the overall small sample sizes limit the comparability between the trial results.

About half of the patients had increases in T cell or antibody magnitudes against multiple antigens post treatment. In addition, a broadening of the antibody repertoire against tumor antigens not covered by BI1361849 was observed after treatment in a subgroup of patients which could be an effect of the radiation, BI1361849 and/or other concomitant cancer treatments resulting in immunogenic cell death of cancer cells. Other studies have shown improved outcomes for cancer patients who respond to multiple TAAs [33–35]. Although these results do not prove causality, it is conceivable that induction of immune responses against multiple TAAs might be of therapeutic benefit.

We observed that the frequency of patients with increased antigen-specific T cells to the individual antigens, in particular CD8^+^ T cells, was rather low. For four of the antigens (NY-ESO-1, 5T4, MAGE-C1, MAGE-C2) fewer than 10% of the treated patients displayed an increase in antigen-specific T cells over baseline. A putative explanation for the low frequencies of tumor antigen-specific T cell responses could be that all of our cellular immune assays were performed ex vivo. Whereas ex vivo assays predominantly assess the functionality of effector T cells, other vaccine antigen-specific T cell subsets including memory T cells may remain undetected [36, 37]. Evidence suggests that central memory T cells which differentiate into effector T cells during the culture period can be detected after prolonged in vitro re-stimulation, whereas circulating effector memory T cells are quantified by ex vivo immune assays. It can be argued that in vitro re-stimulation increases the sensitivity of the assay by increasing the detection of low frequency antigen-specific T-cell responses but there have been reports of inducing de novo priming of naïve T cells during the cell culture. Due to the limited amount of PBMCs available repetition of T cell assays after in vitro stimulation was not possible.

Increases in tumor antigen-specific T cells and antibodies were detected in all three strata suggesting that the different concomitant treatments may not confound this active cancer immunotherapeutic strategy. Elevated antibody responses against cell surface antigens may contribute to antitumor efficacy e.g. through antibody- or complement-dependent cytotoxicity (ADCC, CDC). Radiation may impact the expression of antigens targeted by tumor-specific antibodies [38] and could further enhance the ADCC response in this clinical scenario.

Whereas a putative lack of sensitivity of our ex vivo cellular immune assays could represent a partial explanation for the overall rather low frequency of CD4 and particular CD8 responses, the following factors are probably more relevant and will be addressed in future trials with BI1361849.

Firstly, self-tolerance to the encoded endogenous antigens as well as the presence of immune-suppressive cell populations including myeloid-derived suppressor cells, regulatory T cells, or anti-inflammatory M2 macrophages may counteract the induction of high frequencies of functional T cells. It may be interesting to see if a higher immunogenicity can be achieved in combination with immune checkpoint inhibitors, in particular anti-CTLA4 or anti-PD1/-PD-L1 antibodies, to help break the tolerance against endogenous antigens, e.g. by enhancing effector T cell function and inhibition of regulatory T cells [39]. Also, it may be useful to investigate whether RNActive® immunotherapies encoding patient-specific lung cancer or shared neoantigens might result in higher frequencies of antigen-specific CD8 T cells as suggested by recent studies [40, 41].

Secondly, a critical factor to consider for future trials with protamine-based mRNA immunotherapies is the mode of administration as shown by recent data. A first-in-human trial of a protamine-formulated mRNA-based rabies vaccine showed that needle-syringe injection of this vaccine failed to induce protective virus-neutralizing antibody titers. However, intradermal injection using a needle-free injection device induced antibody titers above the World Health Organization defined protective threshold in 71% of subjects. The serological protection correlated with the induction of multi-functional rabies-specific CD4^+^ T cells assessed with the same ex vivo ICS methodology as described here [42]. Preclinical data showed that needle-free jet injection increases the expression of the mRNA encoded antigens in the skin and improves immunogenicity of protamine formulated mRNA vaccines (CureVac AG, unpublished observations).

Based on these findings, BI1361849 is being investigated in an ongoing phase I/II trial in combination with the anti PD-L1 antibody durvalumab, and the anti CTLA4 antibody tremelimumab and a needle-free injection device is being used for the intradermal injection.

While the design of our phase I trial does not allow us to draw conclusions on the therapeutic benefit of the combination, shrinkage of non-irradiated lesions and prolonged disease stabilization was observed in a subset of patients, mainly in combination with pemetrexed. Two patients on pemetrexed maintenance therapy remained progression free for a period of 21 and 27 months each, and one patient in stratum 3 (EGFR-TKI therapy) in whom a new lesion was detected on follow-up imaging after 14 months of treatment continued BI1361849 for more than 1 year beyond RECIST-defined disease progression. One of the eight patients in stratum 2 who did not receive concomitant maintenance therapy experienced a decrease in target lesion size, but none experienced an objective response.

A limitation of our analysis is the lack of a control group (either radiation, BI1361849, or chemotherapy/EGFR-targeted agent alone), and it is not possible to conclude whether the detected increases in T cells and antibodies observed were solely due to BI361849, radiation or the combination of both. Furthermore, only two patients were recruited to the EGFR-TKI stratum, as most screened patients had no eligible lesions for radiotherapy due to the high response rate to initial therapy.

mRNA-based active cancer immunotherapies, as used in the current study, can be designed to encode for a variety of endogenous cancer antigens from different cancer types. This will help to maximize the chance that an individual patient’s tumor expresses these antigens, and patients with different antigen expression patterns may thus derive benefit from the therapy. The value of targeting neoantigens for cancer immunotherapy has been extensively considered [43–46]. Recently, two studies investigating neoantigen vaccines demonstrated immunogenicity and reported encouraging clinical safety and efficacy data in patients with stage III/IV melanoma in phase I studies [40, 41]. These initial data provide a rationale for testing of neoantigens for active cancer therapy, whereby the combination with radiotherapy and novel therapies such as immune checkpoint inhibitors may further improve the outcomes for cancer patients. Additional research is required to determine how radiotherapy and immunotherapy can be most effectively combined, considering, for instance, optimal fractionation, dose, and the sequencing of radiotherapy and immunotherapy.

## Conclusions

In conclusion, BI1361849 (CV9202) combined with local radiation treatment with or without pemetrexed was well tolerated and antigen-specific immune responses were detected. Available evidence supports further investigation of BI1361849 (CV9202) using needle-free administration technique and combination with immune checkpoint inhibitors.

## Additional files

Additional file 1: Supplementary Materials and Methods. (PDF 465 kb)

Additional file 2: **Table S1.** Peptide sequences of short class-I peptides. (PDF 79 kb)

Additional file 3: **Table S2.** Screening failures. (PDF 250 kb)

Additional file 4: **Table S3.** Treatment exposure during the study. (PDF 322 kb)

Additional file 5: **Table S4.** Treatment-emergent adverse events occurring in ≥10% of patients overall by preferred term (safety analysis set). (PDF 309 kb)

Additional file 6: **Figure S1.** Evaluation of cellular immunity in patients receiving BI1361849 immunotherapy combined with local radiation treatment: representative examples. (PDF 3990 kb)

Additional file 7: **Table S5.** Immune responders per assay at any post-vaccine time point in different strata. (PDF 96 kb)

Additional file 8: **Figure S2.** Heatmap showing broadening of the humoral immune response against antigens in several of the evaluated patients. (PDF 411 kb)

Additional file 9: **Table S6.** Best overall response by lesion type (safety analysis set). (PDF 256 kb)

Additional file 10: **Figure S3.** Survival following BI1361849 immunotherapy combined with local radiation treatment. (PDF 438 kb)

Additional file 11: **Table S7.** Name of the Ethics Committees that approved the study and approval numbers. (PDF 255 kb)

## Abbreviations

5T4: Trophoblast glycoprotein
ADCC: Antibody-dependent cellular cytotoxicity
AE: Adverse events
CDC: Complement-dependent cytotoxicity
CI: Confidence interval
ECOG: Eastern Cooperative Oncology Group
EGFR: Epidermal growth factor receptor
ELISA: Enzyme-linked immunosorbent assay
ELISpot: Enzyme-linked immunosorbent spot
ICS: Intracellular cytokine staining
IFN: Interferon
IL: Interleukin
MAGE-C1/C2: Melanoma antigen family C1/C2
mRNA: Messenger ribonucleic acid
MUC-1: Mucin-1
NCI-CTCAE: National Cancer Institute–Common Terminology Criteria for Adverse Events
NSCLC: Non-small cell lung cancer
NY-ESO-1: New York esophageal squamous cell carcinoma
OS: Overall survival
PBMC: Peripheral blood mononuclear cell
PD-L1: Programmed death-ligand 1
PFS: Progression-free survival
PR: Partial response
RECIST: Response Evaluation Criteria for Solid Tumors
SAF: Safety analysis set
SD: Stable disease
SLD: Sum of longest diameters
TAA: Tumor associated antigens
TEAES: Treatment-emergent AEs
TKI: Tyrosine kinase inhibitor
TLR: Toll-like receptor
TNF-α: Tumor necrosis factor alpha
