# Supplementary material for: Phase Ib evaluation of a self-adjuvanted protamine formulated mRNA-based active cancer immunotherapy, BI1361849 (CV9202), combined with local radiation treatment in patients with stage IV non-small cell lung cancer
Source: J Immunother Cancer. 2019 Feb 8;7:38. doi: 10.1186/s40425-019-0520-5 (PMC6368815; doi:10.1186/s40425-019-0520-5)
Supplement: Supplementary file 1 — Supplementary Materials and Methods. (PDF 465 kb) [file 40425_2019_520_MOESM1_ESM.pdf]

Supplementary Materials and Methods

Tumor lesions eligible for radiation

Eligible tumor lesions included: (a) bone metastases; (b) lymph node metastases in the paraclavicular, axillary or cervical regions; (c) skin or subcutaneous metastases; and (d) for patients in strata 1 and 2 only, thoracic lesions (centrally located lung tumor, lymph node metastases in the lung hilus or mediastinum). If more than one eligible lesion was present, lesions were selected according to the following hierarchy: (a); (b); (c); and (d); the most suitable lesion with minimal expected toxicity effects was chosen for irradiation. Lesions eligible for radiation therapy measured ≥2 cm for lymph node lesions and ≥1 cm for non-lymph node lesions.

Radiation target definition

The treatment volume included the gross tumor volume and a safety margin of 8 mm to create the planning treatment volume; in bone metastases, a clinical target volume margin was added within the involved bone.

Safety assessment

Adverse events (AEs) were collated from the point at which patients gave informed consent through to 30 days after the last dose of study treatment. Beyond this period, AEs considered to be related to the study medication (in particular autoimmune mediated AEs or late radiation pneumonitis) were still required to be reported.

Standard hematological and biochemical assessments were performed at screening, baseline (Day 1), and during the study treatment period. In addition, to monitor for potential autoimmunity, antinuclear antibodies, thyroid stimulating hormone (TSH), antithyroglobulin, antithyroid peroxidase, and anti-TSH receptor antibodies were

evaluated. If patients exhibited TSH levels outside normal ranges, free triiodothyronine and free thyroxine were to be assessed. Physical examinations were also performed.

Assessments and blood sampling for hematological and biochemical tests

Peripheral blood mononuclear cell (PBMC) and serum collection

Venous blood samples for the assessment of humoral and cellular immune responses were collected on Days 1, 19, and 61 and PBMC and sera were prepared. PBMCs were prepared and frozen using a dedicated and standardized kit (CureVac AG, Germany) at a study-related site or local approved laboratory. PBMCs were isolated from sodium-heparin blood Vacutainer tubes (BD Biosciences, Germany) using density centrifugation with Leucosep tubes containing Ficoll-Paque™ PLUS (Greiner Bio-One, Germany). Viable cells were counted, and PBMCs frozen with Cryo-SFM medium (Promocell, Germany) and stored in liquid nitrogen. For preparation of serum samples, blood was collected in Vacutainer serum clot activator 10 mL tubes (BD, Biosciences, Germany). Serum was used for the assessment of antigen-specific humoral immune responses and seromics antibody profiling.

ICS and flow cytometry

Antigen-specific $CD4^+$ and $CD8^+$ T cells were assessed *ex vivo* by ICS measuring interferon-γ (IFN-γ), tumor necrosis factor-α (TNF-α), interleukin-2 (IL-2), and CD107a production by $CD4^+$ and $CD8^+$ T cells. PBMCs were thawed and stimulated *ex vivo* with overlapping 15-mer peptides covering the full open reading frame of the six BI1361849-encoded antigens (JPT Peptide Technologies GmbH, Germany) including literature-selected short class-I peptides. Short-term cell culture was performed for 6 hours in the presence of anti-CD28 (CD28.2), anti-CD49d (L25), PE anti-CD107a (H4A3) antibodies (BD Biosciences, Germany) and GolgiPlug/GolgiStop

(BD Biosciences). Cells stimulated with culture medium X-Vivo 15 (Lonza, Switzerland) + dimethyl sulfoxide (Sigma-Aldrich Chemie GmbH, Germany) served as background controls. In addition, each experimental run included a positive control consisting of PBMCs stimulated with a peptide mix including epitopes from cytomegalovirus, Epstein-Barr virus and flu virus (CEF; JPT Peptide Technologies GmbH, Germany) and Staphylococcus enterotoxin B (SEB) (Sigma-Aldrich Chemie GmbH, Germany). Stimulated PBMCs were incubated with Fc-receptor block (Miltenyi Biotec GmbH, Germany) and labeled with LIVE/DEAD® Fixable Aqua Dead Cell Stain Kit (Thermo Fisher Scientific, USA) and Brilliant Violet650 anti-CD8 (RPA-T8), APC-H7 anti-CD3 (SK7), PE-Cy7 anti-CD4 (MP4-25D2), AlexaFluor700 anti-CD14 (M5E2), or AlexaFluor700 anti-CD19 (HIB19) (BD Biosciences). Cells were fixed and permeabilized using Cytofix/Cytoperm (BD Biosciences) and ICS was performed for the activation marker CD69 (Brilliant Violet605 anti-CD69, clone FN50, BioLegend, Germany) and cytokines using FITC anti-IFN-γ (B27) (BD Biosciences), PerCP-Cy5.5 anti-TNF-α (Mab11), or Brilliant Violet 421 anti-IL-2 (MQ1-17H12) (BioLegend) antibodies.

ICS samples were measured and acquired on a pre-calibrated 3-laser FortessaLSR cytometer using FACSDiva software (BD Biosciences). Compensation controls were prepared with oneComp compensations beads (eBioscience) and corresponding ICS antibodies. Data analysis was performed with FlowJo X software (Treestar Inc, USA). After exclusion of duplets, dead cells, $CD19^+$ B cells, and $CD14^+$ monocytes, $CD3^+$ lymphocytes were gated and further subdivided in $CD4^+$ and $CD8^+$ T cells. The

proportion of activated, cytokine-producing cells within $CD4^+$ and $CD8^+$ T cell populations was determined.

Ex vivo IFN-γ ELISpot

PBMCs were thawed and rested overnight. Afterwards the cells were stimulated with overlapping 15-mer peptides covering the full open reading frame of the six BI1361849-encoded antigens (JPT Peptide Technologies GmbH, Germany) and spiked short literature-based class-I peptides for 24 hours. PBMCs cultured in the presence of CEF and SEB were used as controls. The number of IFN-γ-producing cells was measured by ELISpot and analyzed according to recommendations of the Cancer Immunotherapy Consortium and the Association for Cancer Immunotherapy.

BI1361849-specific immunoglobulin M (IgM) and immunoglobulin G (IgG) antibodies in sera

BI1361849-specific IgG and IgM antibody levels in blood sera were measured by ELISA by Serametrix (UK). Each serum sample was first subjected to a serial three-fold dilution in sample buffer: 1:3, 1:9, 1:27, and 1:81. Two aliquots (100 µL) from each dilution were then added to ELISA plates containing full-length human recombinant proteins corresponding to each of the BI1361849-encoded antigens. Bound serum antibody was detected using a secondary antibody conjugate with colorimetric readout using a standard ELISA plate reader (Molecular Devices) set to detect optical density (OD) at 450nm (OD450). For each dilution for each antigen, the data pair was averaged to produce a raw data set reporting the signal for each antigen sample across all four dilutions.

Serametrix serum profiling assay (microarray)

To investigate change in serum antibody patterns, patient serum samples were diluted (1:20) in sample buffer and exposed to the Lung Cancer Antigen Microarray

(Serametrix), containing 33 full-length human tumor associated antigens (TAAs) immobilized onto a planar solid support medium (see table below).

Table: Tumor associated antigens (TAAs) Serametrix lung cancer panel

5T4*	LDHC	NXF2	SSX2
CABYR	MAGEA3	NYESO1*	SSX4
CALR3 (CT93)	MAGEA4	p53	SSX5
COX6B2 (CT59)	MAGEA5	PBK	Survivin*
CSAG2	MAGEB1	PRAME	TPTE
CTAG2 Homolog	MAGEC1*	PTTG1	TSGA10
FATE1 (CT43)	MAGEC2*	SPANXA1	XAGE2
FTHL17	MUC-1*	SSX1	ZNF165

* BI1361849-associated antigens (TAAs)

The array was then washed before being probed with secondary antibody (mouse anti-human-IgG Cy-5 conjugate at 1:400) in order to detect bound serum antibody. Arrays were scanned at 635nm using a microarray scanner (Molecular Devices) and data recorded as Relative Fluorescent Units (RFU). Signals greater than threshold were reported as positive. The threshold value was derived from background, where background = [2.5] x [1st quartile] (Gnjactic et al. 2009). The first quartile was calculated from all samples with all antigens. The number of positive responses against antigens was recorded as the 'score' for each sample. The number of responses against different TAAs were calculated for each time point. The number of accrual responses corresponds to the baseline subtracted 'score'.

Efficacy assessments

Radiological tumor assessments — computed tomography or magnetic resonance imaging scans of the thorax, abdomen, and other tumor-bearing regions, as applicable — were performed 1–2 weeks before starting study treatment for patients in strata 1 and 2, or in the 4 weeks before starting study treatment for stratum 3. Baseline bone scans were performed for patients with bone metastases. In

accordance with standard NSCLC practice, further radiological tumor assessments were performed every 6 weeks during the treatment period, using the same imaging technique, if possible.

After completing study treatment, all patients were followed up for survival and response to subsequent systemic cancer therapy; follow-up took place every 3 months until death, withdrawal of informed consent for, or loss to, follow-up. Patients were followed-up for up to 18 months after the start of treatment for the last patient enrolled.

Immune data analysis

In addition to the pre-specified definitions for cellular and humoral immunomonitoring described in the Methods of the manuscript, further empirical rules for definitions immune responses were applied, as detailed below.

For ICS assay, the measured cytokine response was required to equal or exceed a frequency of 0.01% or alternatively 10 events in the gate. Two raters independently confirmed the data by visual inspection (four-eye optical rating) of the flow cytometry plots, to exclude possible artefacts.

ELISpot required a pre-specified minimal spot count (≥10 spots/well) measured at least as duplicate values, with non-overlapping error bars for respective comparisons (e.g. baseline vs post-administration of BI1361849). All defined positive ELISpot results were further examined, inspecting the original ELISpot counter-derived data to exclude potential artefacts (e.g. false positive counts).

For IgM and IgG antibody ELISA, definitions were further based on the optimal OD; ODs had to be higher than the negative control and the dilution yielding the closest

OD to 0.5 was chosen to obtain comparative data in the linear range. Background subtraction limited matrix effects. All responses were examined to exclude artefacts (e.g. false positive results due to irregularities in assay performance).

In addition to analyzing analytical method-specific data for each of the separate BI1361849-encoded antigens, broader statistical categories were used to assess post-baseline effects against at least one of the six BI1361849 antigens for T cells, measured by ICS (antigen specific $CD4^+$ and/or $CD8^+$ T cells) and ELISpot (IFN-γ secreting cells), humoral effects , whereby antigen-specific IgM and IgG antibodies were measured via ELISA, and overall effects, recorded using any of the analytical methods (ELISpot, ICS, and/or ELISA).

Reference

Gnjactic S, Wheeler C, Ebner M, et al. Seromic analysis of antibody responses in non-small cell lung cancer patients and healthy donors using conformational protein arrays. J Immunol Methods 2009;341:50–8.
