# Supplementary material for: Phase Ib evaluation of a self-adjuvanted protamine formulated mRNA-based active cancer immunotherapy, BI1361849 (CV9202), combined with local radiation treatment in patients with stage IV non-small cell lung cancer
Source: J Immunother Cancer. 2019 Feb 8;7:38. doi: 10.1186/s40425-019-0520-5 (PMC6368815; doi:10.1186/s40425-019-0520-5)
Supplement: Supplementary file 2 — Table S1. Peptide sequences of short class-I peptides. (PDF 79 kb) [file 40425_2019_520_MOESM2_ESM.pdf]

Table S1. Peptide sequences of short class-I peptides.

	Antigen name					
	**NY-ESO-1**	**MAGE-C1**	**MAGE-C2**	**5T4**	**Survivin**	**MUC-1**
**Peptide sequence**	SLLMWITQC	ILFGISLREV	LLFGLALIEV	LTYVSFRNL	ELTLGEFLKL	STAPPVHNV
	MLMAQEALAFL	KVVEFLAML	ALKDVEERV	GAFEHLPSL	RISTFKNWPFL	LLLLTVLTV
	LMAQEALAFL		FLAKLNNTV	DLPAYVRNL	FLKLDRERA	STAPPAHGV
	ASGPGGGAPR		SESIKKKVL	RLARLALVL	AYACNTSTL	TLAPATEPA
	LAAQERRVPR		ASSTLYLVF	FLTGNQLAV	FFCFKELEGW	
	TVSGNILTIR		KVAELVEFL	PLADLSPFA	EPDLAQCFF	
	ELVRRILSR				CPTENEPDL	
	APRGPHGGAASGL					
	APRGVRMAV					
	MPFATPMEA					
	KEFTVSGNILTI					
	MPFATPMEA					
	LAMPFATPM					
	ARGPESRLL					
	LEFYLAMPF					
