# Supplementary material for: Phase Ib evaluation of a self-adjuvanted protamine formulated mRNA-based active cancer immunotherapy, BI1361849 (CV9202), combined with local radiation treatment in patients with stage IV non-small cell lung cancer
Source: J Immunother Cancer. 2019 Feb 8;7:38. doi: 10.1186/s40425-019-0520-5 (PMC6368815; doi:10.1186/s40425-019-0520-5)
Supplement: Supplementary file 3 — Table S2. Screening failures. (PDF 250 kb) [file 40425_2019_520_MOESM3_ESM.pdf]

Table S2. Screening failures.

Center	Subject ID	Age (yrs)	Gender	Race	Subject treated?	Discontinuation date
102	102001	50	Female	White	No	03JUL2014
104	104001	61	Male	White	No	08OCT2014
	104002	53	Male	White	No	03NOV2014
301	301004	71	Male	White	No	21OCT2013
304	304001	65	Female	White	No	14JAN2014
	304003	78	Female	White	No	01OCT2014
