# Supplementary material for: Phase Ib evaluation of a self-adjuvanted protamine formulated mRNA-based active cancer immunotherapy, BI1361849 (CV9202), combined with local radiation treatment in patients with stage IV non-small cell lung cancer
Source: J Immunother Cancer. 2019 Feb 8;7:38. doi: 10.1186/s40425-019-0520-5 (PMC6368815; doi:10.1186/s40425-019-0520-5)
Supplement: Supplementary file 4 — Table S3. Treatment exposure during the study. (PDF 322 kb) [file 40425_2019_520_MOESM4_ESM.pdf]

Table S3. Treatment exposure during the study.

Characteristic	Stratum 1 (n=16)	Stratum 2 (n=8)	Stratum 3 (n=2)	Overall (n=26)
BI1361849				
Successful administrations, n				
Median (range)	7 (2–25)	7 (4–11)	15 (5–25)	7 (2–25)
Treatment duration, days				
Median (range)	99 (8–806)	79 (29–163)	425 (57–793)	81 (8–806)
Radiation				
Total dose, Gy				
Median (range)	20 (20–20)	20 (20–40[a])	20 (20–20)	20 (20–40[a])
Pemetrexed				
Treatment duration, days				
Median (range)	45 (1–778)	—	—	—
EGFR TKI				
Dose adjustments, n (%)	—	—	1 (50)	—

[a]In one patient, two lesions were irradiated with 4 × 5 Gy on the same days; total exposure 20, not 40, Gy, as recorded in the database, results for which are shown above.
Abbreviations: TKI, tyrosine kinase inhibitor.
