# Supplementary material for: Phase Ib evaluation of a self-adjuvanted protamine formulated mRNA-based active cancer immunotherapy, BI1361849 (CV9202), combined with local radiation treatment in patients with stage IV non-small cell lung cancer
Source: J Immunother Cancer. 2019 Feb 8;7:38. doi: 10.1186/s40425-019-0520-5 (PMC6368815; doi:10.1186/s40425-019-0520-5)
Supplement: Supplementary file 5 — Table S4. Treatment-emergent adverse events occurring in ≥10% of patients overall by preferred term (safety analysis set). (PDF 309 kb) [file 40425_2019_520_MOESM5_ESM.pdf]

Table S4. Treatment-emergent adverse events occurring in ≥10% of patients overall by preferred term (safety analysis set).

	Number (%) of patients			
**Preferred term**	**Stratum 1 (n=16)**	**Stratum 2 (n=8)**	**Stratum 3 (n=2)**	**Overall (n=26)**
Patients with at least 1 TEAE	16 (100.0)	8 (100.0)	2 (100.0)	26 (100.0)
Injection site erythema	11 (68.8)	7 (87.5)	1 (50.0)	19 (73.1)
Pyrexia	9 (56.3)	4 (50.0)	2 (100.0)	15 (57.7)
Decreased appetite	8 (50.0)	4 (50.0)	0	12 (46.2)
Fatigue	7 (43.8)	3 (37.5)	2 (100.0)	12 (46.2)
Cough	5 (31.3)	4 (50.0)	0	9 (34.6)
Nausea	3 (18.8)	4 (50.0)	2 (100.0)	9 (34.6)
Vomiting	5 (31.3)	1 (12.5)	1 (50.0)	7 (26.9)
Chills	5 (31.3)	1 (12.5)	1 (50.0)	7 (26.9)
Influenza like illness	4 (25.0)	2 (25.0)	1 (50.0)	7 (26.9)
Anemia	6 (37.5)	0	0	6 (23.1)
Dyspnea	4 (25.0)	1 (12.5)	0	5 (19.2)
Arthralgia	2 (12.5)	1 (12.5)	1 (50.0)	4 (15.4)
Constipation	3 (18.8)	1 (12.5)	0	4 (15.4)
Dizziness	2 (12.5)	1 (12.5)	1 (50.0)	4 (15.4)
Injection site pain	2 (12.5)	2 (25.0)	0	4 (15.4)
Edema peripheral	4 (25.0)	0	0	4 (15.4)
General physical health deterioration	1 (6.3)	0	2 (100.0)	3 (11.5)
Injection site discoloration	2 (12.5)	0	1 (50.0)	3 (11.5)
Injection site rash	3 (18.8)	0	0	3 (11.5)
Injection site reaction	3 (18.8)	0	0	3 (11.5)
Neutrophil count decreased	3 (18.8)	0	0	3 (11.5)
Myalgia	3 (18.8)	0	0	3 (11.5)
Headache	2 (12.5)	0	1 (50.0)	3 (11.5)
Insomnia	1 (6.3)	2 (25.0)	0	3 (11.5)
Pruritus	2 (12.5)	1 (12.5)	0	3 (11.5)
Rash	2 (12.5)	0	1 (50.0)	3 (11.5)

Abbreviation: TEAE, treatment-emergent adverse event.
