# Supplementary material for: Phase Ib evaluation of a self-adjuvanted protamine formulated mRNA-based active cancer immunotherapy, BI1361849 (CV9202), combined with local radiation treatment in patients with stage IV non-small cell lung cancer
Source: J Immunother Cancer. 2019 Feb 8;7:38. doi: 10.1186/s40425-019-0520-5 (PMC6368815; doi:10.1186/s40425-019-0520-5)
Supplement: Supplementary file 6 — Figure S1. Evaluation of cellular immunity in patients receiving BI1361849 immunotherapy combined with local radiation treatment: representative examples. (PDF 3990 kb) [file 40425_2019_520_MOESM6_ESM.pdf]

Figure S1. Evaluation of cellular immunity in patients receiving BI1361849 immunotherapy combined with local radiation treatment: representative examples.

(a) Representative example of a positive ex vivo IFN-γ ELISpot assay in a cellular immune responder. Numbers above the images show the number of IFN-γ producing cells per 3×10^5 cells. (b) Representative example of the gating strategy used in *ex vivo* ICS determination of antigen-specific cytokine production. (c) Representative example of changes in antigen-specific $CD8^+$ T cells post-vaccine, as determined *ex vivo* via ICS for NY-ESO-1 specific T cells, with the numbers of IFN-γ, TNF-α, IL-2, or CD107a-positive cells shown in the boxes.

a.

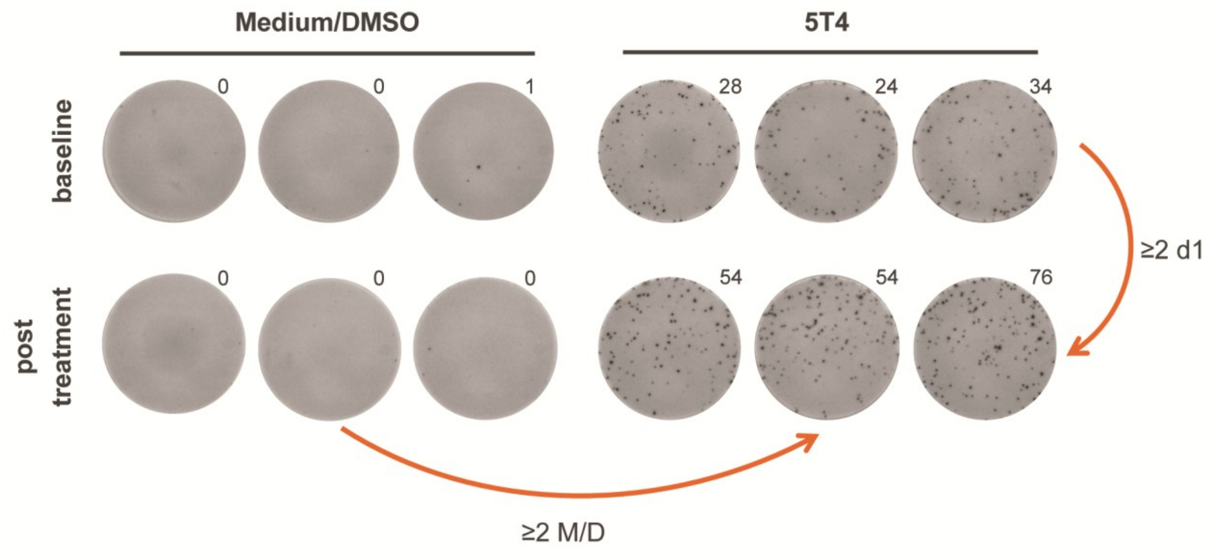

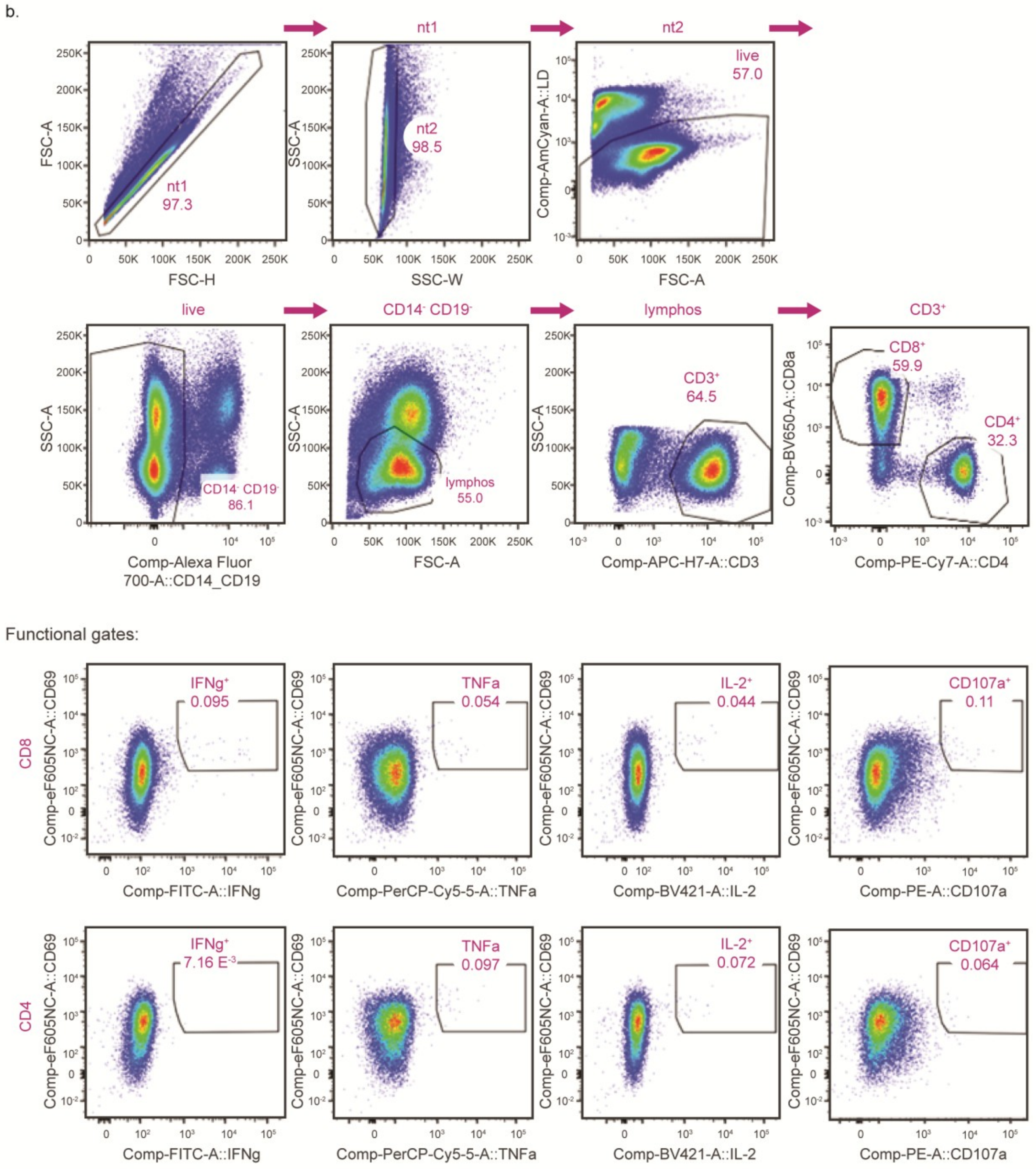
b.
nt1
nt2
live
nt1
97.3
nt2
98.5
live
57.0
FSC-A
FSC-H
SSC-A
SSC-W
Comp-AmCyan-A::LD
FSC-A
live
CD14- CD19-
lymphos
CD3+
CD14- CD19-
86.1
lymphos
55.0
CD3+
64.5
CD8+
59.9
CD4+
32.3
Comp-Alexa Fluor 700-A::CD14_CD19
FSC-A
Comp-APC-H7-A::CD3
Comp-PE-Cy7-A::CD4
Comp-BV650-A::CD8a
Functional gates:
CD8
IFNg+
0.095
TNFa
0.054
IL-2+
0.044
CD107a+
0.11
CD4
IFNg+
7.16 E-3
TNFa
0.097
IL-2+
0.072
CD107a+
0.064
Comp-eF605NC-A::CD69
Comp-FITC-A::IFNg
Comp-PerCP-Cy5-5-A::TNFa
Comp-BV421-A::IL-2
Comp-PE-A::CD107a

c.

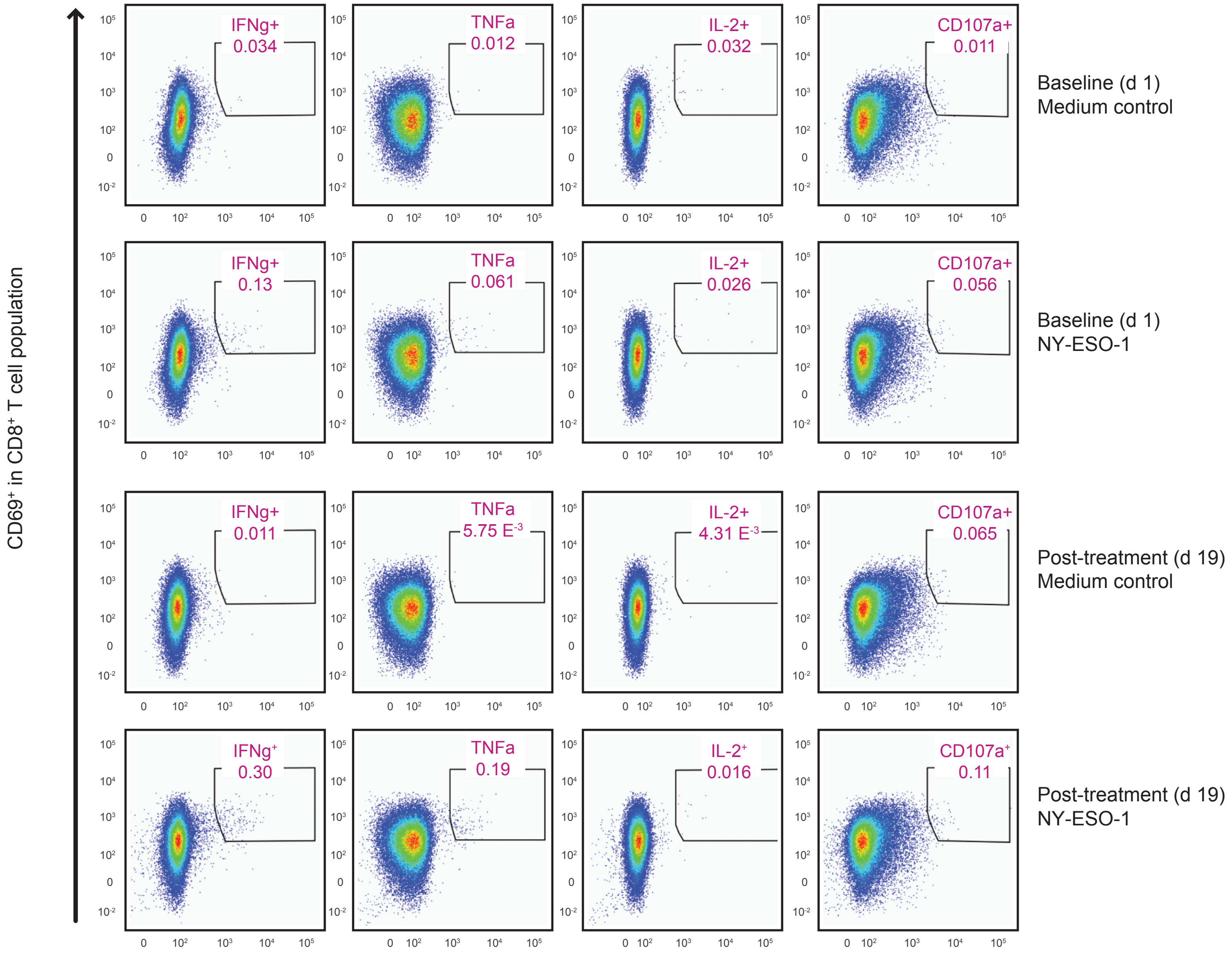
CD69+ in CD8+ T cell population
IFNg+ 0.034
TNFa 0.012
IL-2+ 0.032
CD107a+ 0.011
Baseline (d 1) Medium control
IFNg+ 0.13
TNFa 0.061
IL-2+ 0.026
CD107a+ 0.056
Baseline (d 1) NY-ESO-1
IFNg+ 0.011
TNFa 5.75 E-3
IL-2+ 4.31 E-3
CD107a+ 0.065
Post-treatment (d 19) Medium control
IFNg+ 0.30
TNFa 0.19
IL-2+ 0.016
CD107a+ 0.11
Post-treatment (d 19) NY-ESO-1
