# Supplementary material for: Phase Ib evaluation of a self-adjuvanted protamine formulated mRNA-based active cancer immunotherapy, BI1361849 (CV9202), combined with local radiation treatment in patients with stage IV non-small cell lung cancer
Source: J Immunother Cancer. 2019 Feb 8;7:38. doi: 10.1186/s40425-019-0520-5 (PMC6368815; doi:10.1186/s40425-019-0520-5)
Supplement: Supplementary file 7 — Table S5. Immune responders per assay at any post-vaccine time point in different strata. (PDF 96 kb) [file 40425_2019_520_MOESM7_ESM.pdf]

Table S5. Immune responders per assay at any post-baseline time point in different strata.

Stratum	First-line treatment	ELISpot	ICS			IgM	IgG	Microarray*	Cellular	Humoral	Overall
			$CD4^+$ T cells	$CD8^+$ T cells	$CD4^+$or $CD8^+$ T cells						
**1**	Platinum + pemetrexed	2/7 (28.6)	5/15 (33.3)	2/15 (13.3)	6/15 (40.0)	5/15 (33.3)	14/15 (93.3)	8/14 (57.1)	7/15 (46.7)	14/15 (93.3)	14/15 (93.3)
**2**	Platinum-based	0/3 (0)	1/8 (12.5)	2/8 (25.0)	2/8 (25.0)	1/8 (12.5)	5/8 (62.5)	2/8 (25.0)	2/8 (25.0)	5/8 (62.5)	6/8 (75.0)
**3**	EGFR TKI	0/0 (0)	1/2 (50.0)	0/2 (0)	1/2 (50.0)	1/2 (50.0)	0/2 (50.0)	2/2 (100)	1/2 (50.0)	1/2 (50.0)	1/2 (50.0)

The data displayed in the body of the table show the numbers of responders/the total numbers of patient samples analyzed (percentage responders). Cellular immune responses were determined ex vivo by ELISpot assay and ICS. Antigen-specific antibodies were determined by enzyme-linked immunosorbent assay (ELISA). Definitions of immune responders, both cellular and humoral, included criteria of two-fold increases in immune response criteria over baseline (Day 1) as well as over background.
Abbreviations: TKI, tyrosine kinase inhibitor; ELISpot, enzyme-linked immunosorbent spot; Ig, immunoglobulin; ICS, intracellular cytokine staining

* Seromic profiling, using the Serametrix NSCLC-specific antigen microarray, was performed to investigate broadening of humoral immunity. The numbers displayed refer to patients with broadening of the antibody repertoire against antigens not covered by BI1361849/total number of patients analysed.
