# Supplementary material for: Phase Ib evaluation of a self-adjuvanted protamine formulated mRNA-based active cancer immunotherapy, BI1361849 (CV9202), combined with local radiation treatment in patients with stage IV non-small cell lung cancer
Source: J Immunother Cancer. 2019 Feb 8;7:38. doi: 10.1186/s40425-019-0520-5 (PMC6368815; doi:10.1186/s40425-019-0520-5)
Supplement: Supplementary file 8 — Figure S2. Heatmap showing broadening of the humoral immune response against antigens in several of the evaluated patients. (PDF 411 kb) [file 40425_2019_520_MOESM8_ESM.pdf]

Figure S2. Heatmap showing broadening of the humoral immune response against antigens in several of the evaluated patients.

Patient serum samples were exposed to the Lung Cancer Antigen Microarray (Serametrix). Arrays were scanned at 635 nm using a microarray scanner (Molecular Devices) and data recorded as Relative Fluorescent Units. Signals greater than threshold were reported as positive. The threshold value was derived from background, where background = [2.5] x [1st quartile]. Heat maps showing, for each time point and each patient, whether the patient had a signal higher than the 17230 threshold (orange fill). Antigens names covered by BI1361849 are displayed in blue/bold.

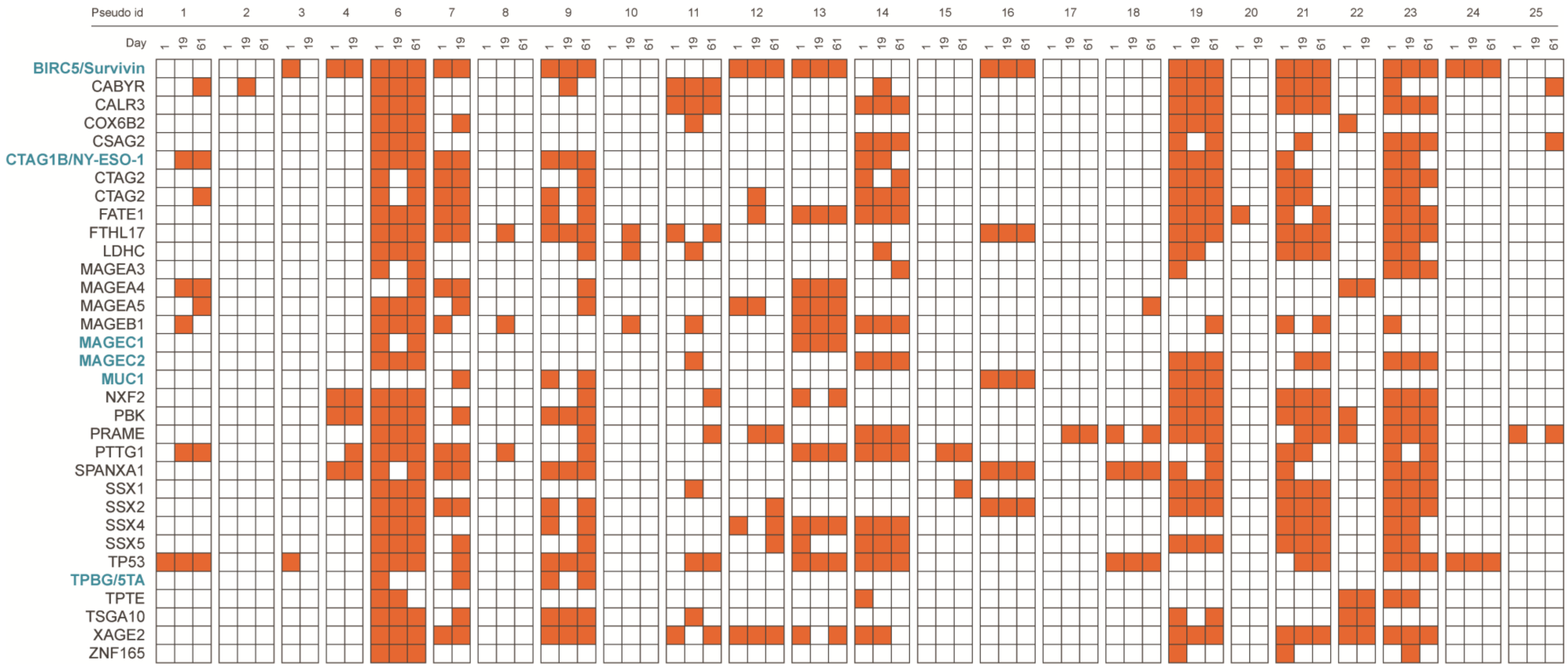
Pseudo id
1
2
3
4
6
7
8
9
10
11
12
13
14
15
16
17
18
19
20
21
22
23
24
25
Day
1
19
61
BIRC5/Survivin
CABYR
CALR3
COX6B2
CSAG2
CTAG1B/NY-ESO-1
CTAG2
CTAG2
FATE1
FTHL17
LDHC
MAGEA3
MAGEA4
MAGEA5
MAGEB1
MAGEC1
MAGEC2
MUC1
NXF2
PBK
PRAME
PTTG1
SPANXA1
SSX1
SSX2
SSX4
SSX5
TP53
TPBG/5TA
TPTE
TSGA10
XAGE2
ZNF165
