# Supplementary material for: Phase Ib evaluation of a self-adjuvanted protamine formulated mRNA-based active cancer immunotherapy, BI1361849 (CV9202), combined with local radiation treatment in patients with stage IV non-small cell lung cancer
Source: J Immunother Cancer. 2019 Feb 8;7:38. doi: 10.1186/s40425-019-0520-5 (PMC6368815; doi:10.1186/s40425-019-0520-5)
Supplement: Supplementary file 9 — Table S6. Best overall response by lesion type (safety analysis set). (PDF 256 kb) [file 40425_2019_520_MOESM9_ESM.pdf]

Table S6. Best overall response by lesion type (safety analysis set).

	Patients with response, n (%)<br>[95% confidence interval]			
	**Stratum 1<br>(n=16)**	**Stratum 2<br>(n=8)**	**Stratum 3<br>(n=2)**	**Overall<br>(n=26)**
Target lesions				
CR	1 (6.3)<br>[0.16, 30.23]	0<br>[0.00, 36.94]	0<br>[0.00, 84.19]	1 (3.8)<br>[0.10, 19.64]
PR	0<br>[0.00, 20.59]	0<br>[0.00, 36.94]	0<br>[0.00, 84.19]	0<br>[0.00, 13.23]
SD	10 (62.5)<br>[35.43, 84.80]	4 (50.0)<br>[15.70, 84.30]	1 (50.0)<br>[1.26, 98.74]	15 (57.7)<br>[36.92, 76.65]
PD	3 (18.8)<br>[4.05, 45.65]	2 (25.0)<br>[3.19, 65.09]	1 (50.0)<br>[1.26, 98.74]	6 (23.1)<br>[8.97, 43.65]
NE	2 (12.5)<br>[1.55, 38.35]	2 (25.0)<br>[3.19, 65.09]	0<br>[0.00, 84.19]	4 (15.4)<br>[4.36, 34.87]
Non-target lesions				
CR	1 (6.3)<br>[0.16, 30.23]	0<br>[0.00, 36.94]	0<br>[0.00, 84.19]	1 (3.8)<br>[0.10, 19.64]
Non-CR/non-PD	10 (62.5)<br>[35.43, 84.80]	6 (75.0)<br>[34.91, 96.81]	1 (50.0)<br>[1.26, 98.74]	17 (65.4)<br>[44.33, 82.79]
PD	4 (25.0)<br>[7.27, 52.38]	1 (12.5)<br>[0.32, 52.65]	1 (50.0)<br>[1.26, 98.74]	6 (23.1)<br>[8.97, 43.65]
NE	1 (6.3)<br>[0.16, 30.23]	1 (12.5)<br>[0.32, 52.65]	0<br>[0.00, 84.19]	2 (7.7)<br>[0.95, 25.13]

Non-CR/non-PD: persistence of one or more non-target lesion(s) or/and maintenance of tumor markers (carcinoembryonic antigen, cytokeratin fragment 21-1) above the normal limits.
Abbreviations: CR, complete response; NE, not evaluable; PD, progressive disease; PR, partial response; SD, stable disease
