# Supplementary material for: Phase Ib evaluation of a self-adjuvanted protamine formulated mRNA-based active cancer immunotherapy, BI1361849 (CV9202), combined with local radiation treatment in patients with stage IV non-small cell lung cancer
Source: J Immunother Cancer. 2019 Feb 8;7:38. doi: 10.1186/s40425-019-0520-5 (PMC6368815; doi:10.1186/s40425-019-0520-5)
Supplement: Supplementary file 10 — Figure S3. Survival following BI1361849 immunotherapy combined with local radiation treatment. (PDF 438 kb) [file 40425_2019_520_MOESM10_ESM.pdf]

Figure S3. Survival following BI1361849 immunotherapy combined with local radiation treatment.

(a) Kaplan–Meier progression-free survival curve (safety analysis set). (b) Kaplan–Meier overall survival curve (safety analysis set).

a.

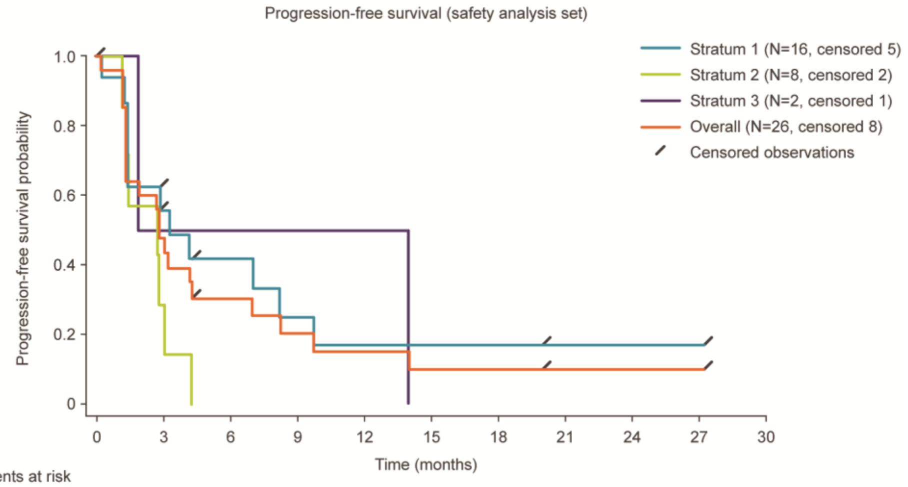

Patients at risk											
Stratum 1	16	8	5	3	2	2	2	1	1	1	0
Stratum 2	8	2	0	0	0	0	0	0	0	0	0
Stratum 3	2	1	1	1	1	0	0	0	0	0	0
Overall	26	11	6	4	3	2	2	1	1	1	0

b.

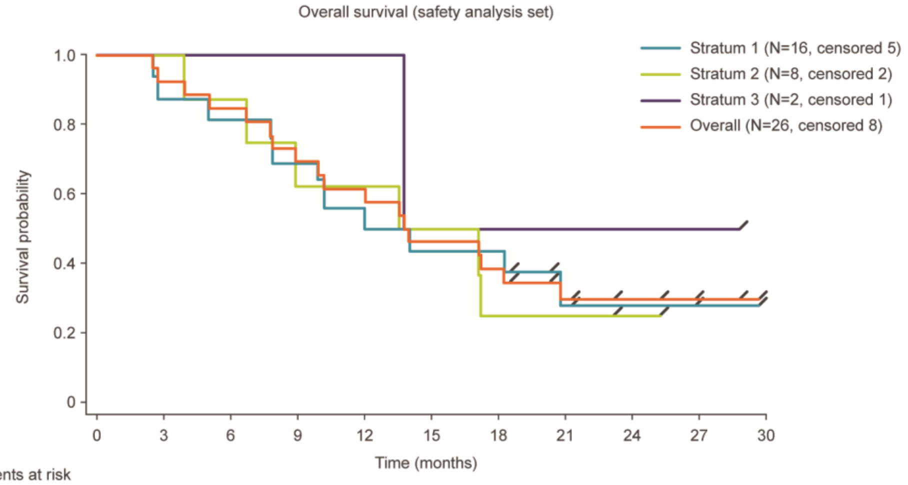

Patients at risk											
Stratum 1	16	14	13	11	9	7	7	3	2	1	0
Stratum 2	8	8	7	5	5	4	2	2	1	0	0
Stratum 3	2	2	2	2	2	1	1	1	1	1	0
Overall	26	24	22	18	16	12	10	6	4	2	0
