# Supplementary material for: Phase Ib evaluation of a self-adjuvanted protamine formulated mRNA-based active cancer immunotherapy, BI1361849 (CV9202), combined with local radiation treatment in patients with stage IV non-small cell lung cancer
Source: J Immunother Cancer. 2019 Feb 8;7:38. doi: 10.1186/s40425-019-0520-5 (PMC6368815; doi:10.1186/s40425-019-0520-5)
Supplement: Supplementary file 11 — Table S7. Name of the Ethics Committees that approved the study and approval numbers. (PDF 255 kb) [file 40425_2019_520_MOESM11_ESM.pdf]

Table S7. Name of the Ethics Committees that approved the study and approval numbers.

Ethics committee name	Function	EC Ref No.
**Switzerland**		
Ethikkommission beider Basel (later: Ethikkommission Nordwest- und Zentralschweiz)	Leading EC	284/12
Kantonale Ethikkommission (KEK) Universitätsspital Zürich	Local EC	KEK-ZH-Nr. 2013-0093
Ethikkommission Kantonsspital St. Gallen	Local EC	EKSG 12/178
Kantonale Ethikkommission (KEK)	Local EC	KEK-ZH-Nr. 2013-0143
**Austria**		
Ethikkommission der Medizinischen Universität Innsbruck	Leading EC	UN4898_LEK;320/2.2, 321/2.4; AN4898
**Germany**		
Ethik-Kommission des Fachbereichs Medizinder Johann-Wolfgang-Goethe-Universität Frankfurt am Main	Leading EC	396/12
Ethik-Kommission der Landesärztekammer Rheinland-Pfalz	Local EC	837.509.12; (8610)
Ethik-Kommission bei der Landesärztekammer Baden-Württemberg	Local EC	B-AM-201-034 A3
Ethik-Kommission des Landes BerlinLandesamt für Gesundheit und Soziales	Local EC	14/118 EK 15
Ethik-Kommission bei der Ärztekammer Niedersachsen Unterkommission zur Beurteilung med. Forschung am Menschen	Local EC	No own number (via leading EC)
Ethik-Kommission der Ärztekammer Nordrhein	Local EC	2014106
Ethik-Kommission der Ärztekammer Westfalen-Lippe und der mediz. Fakultät der Westfälischen Wilhelms Universität Münster	Local EC	2014-158-b-A
Ethik-Kommission der medizinischen Fakultät Heidelberg	Local EC	No own number (via leading EC)
